# Clinic-based evaluation of point-of-care dual HIV/syphilis rapid diagnostic tests at primary healthcare antenatal facilities in South Africa and Zambia

**DOI:** 10.1186/s12879-024-09463-1

**Published:** 2024-06-19

**Authors:** Ranmini Kularatne, Karel Blondeel, Margaret Kasaro, Venessa Maseko, Samuel Bosomprah, Ronaldo Silva, Maura Laverty, Firdavs Kurbonov, Massimo Mirandola, Rosanna W Peeling

**Affiliations:** 1 Centre for HIV & STI, National Institute for Communicable Diseases, Johannesburg, South Africa; 2 Department of Clinical Microbiology & Infectious Diseases, Faculty of Health Sciences, University of the Witwatersrand, Johannesburg, South Africa; 3 Faculty of Medicine and Health Sciences, Ghent University, Ghent, Belgium; 4 University of North Carolina Global Projects Zambia, Lusaka, Zambia; 5 Department of Obstetrics and Gynecology, School of Medicine, University of North Carolina, Chapel Hill, USA; 6 Center for Infectious Diseases Research in Zambia, Lusaka, Zambia; 7 Department of Biostatistics, School of Public Health, University of Ghana, Legon, Accra Ghana; 8 Negrar di Valpolicella (VR), Verona, Italy; 9 Greenwich, USA; 10 Dushanbe, Tajikistan; 11 Department of Diagnostics and Public Health, Infectious Diseases Section, University of Verona, Verona, Italy; 12 Department of Clinical Research, London School of Hygiene and Tropical Medicine, Verona, UK

**Keywords:** Point-of-care tests, HIV, Syphilis, Dual EMTCT HIV/syphilis, Public health, Diagnostic evaluation, Clinic-based evaluation, ANC, Antenatal care

## Abstract

**Background:**

Southern African countries have the largest global burden of HIV and syphilis, with a high prevalence among women of reproductive age. Although antenatal screening is standard of care, syphilis screening has generally lagged behind HIV screening. We aimed to evaluate the performance and operational characteristics of two commercial dual HIV/syphilis point-of-care tests (POCTs) for simultaneous maternal HIV/syphilis screening.

**Methods:**

A clinic-based evaluation of dual HIV/syphilis POCTs (SD Bioline and Chembio) was conducted at five primary healthcare centres (PHCs) in South Africa and Zambia. POCT results using capillary fingerprick blood were compared to reference laboratory syphilis and HIV serological assays.

**Results:**

Three thousand four hundred twelve consenting pregnant women aged ≥ 18 years were enrolled. The prevalence of treponemal antibody seropositivity and HIV infection ranged from 3.7 to 9.9% (*n* = 253) and 17.8 to 21.3% (*n* = 643), respectively. Pooled sensitivity for syphilis compared to the reference assay was 66.0% (95%CI 57.7–73.4) with SD Bioline and 67.9% (95%CI 58.2–76.3) with Chembio. Pooled specificity for syphilis was above 98% with both POCTs. The sensitivities of SD Bioline and Chembio assays were 78.0% (95%CI 68.6–85.7) and 81.0% (95%CI 71.9–88.2), respectively compared to an active syphilis case definition of treponemal test positive with a rapid plasma reagin titre of ≥ 8. The negative predictive values (NPVs) based on various prevalence estimates for syphilis with both assays ranged from 97 to 99%. The pooled sensitivity for HIV was 92.1% (95%CI 89.4–94.2) with SD Bioline; and 91.5% (95%CI 88.2–93.9) with Chembio. The pooled specificities for HIV were 97.2% (95%CI 94.8–98.5) with SD Bioline and 96.7% (95%CI 95.1–97.8) with Chembio. The NPV based on various prevalence estimates for HIV with both assays was approximately 98%. Most participating women (91%) preferred dual POCTs over two single POCTs for HIV and syphilis, and healthcare providers gave favourable feedback on the utility of both assays at PHC level.

**Conclusions:**

Based on the need to improve antenatal screening coverage for syphilis, dual HIV/syphilis POCTs could be effectively incorporated into antenatal testing algorithms to enhance efforts towards elimination of mother-to-child transmission of these infections.

**Supplementary Information:**

The online version contains supplementary material available at 10.1186/s12879-024-09463-1.

## Introduction

The Southern African region bears the largest global burden of HIV and syphilis. In South Africa, there were an estimated 7.8 million people living with HIV in 2020 [1]. When population-level data were stratified by age and gender, women aged 15–49 years had the highest incidence and prevalence of HIV in the 10-year period between 2010 and 2020 [1, 2]. The most recent South African antenatal survey was conducted in 2019 among approximately 37,000 pregnant women across all 9 provinces of the country [3]. The national antenatal HIV prevalence was estimated at 30% (95%CI 29.4–30.6) and has remained stable since the previous survey undertaken in 2017. However, the rapid plasma reagin (RPR) positivity for antenatal syphilis seroprevalence had increased by 30% from 2.0% at the time of the last syphilis serosurvey in 2015 to 2.6% (95%CI 2.4–2.9) in 2019 [3]. In Zambia, in 2018 the HIV prevalence among women of reproductive age was estimated at 14.2% (95%CI 13.1–15.3) [4], while nationally reported maternal syphilis prevalence was 5% [5].

Rapid HIV tests are routinely utilised for HIV screening in primary healthcare settings in South Africa to give on-site same-day results and facilitate the universal HIV test-and-treat policy that was implemented in 2016. The national HIV screening algorithm recommends a single HIV point-of-care test (POCT) followed, if positive, by confirmation with a second different HIV POCT [6]. Despite having a national recommendation, syphilis screening and treatment coverage rates have lagged behind those for HIV in antenatal care [7]. This is because syphilis testing has largely utilised laboratory-based treponemal and non-treponemal assays which require serum or plasma instead of whole blood, longer turnaround times and follow-up visits for results. WHO guidelines recommend HIV and syphilis screening early in pregnancy, at the first antenatal visit; further maternal retesting is particularly advised for HIV, and may include the use of the dual HIV/syphilis POCTs, in the 3rd trimester and catch-up testing at the earliest possible time point for women who miss or present late [8]. In South Africa, syphilis screening is recommended at the first antenatal clinic visit, preferably in the first trimester of pregnancy but approximately 60% of pregnant women present to antenatal care in the second trimester [3]. Additionally, because these women are deemed to be at high-risk, given the relatively high HIV prevalence, national policy mandates a second screening test for syphilis at 32–34 weeks, if the first trimester result is negative [9]. These factors highlight the requirement for a rapid syphilis POCT that can be performed simultaneously with HIV testing.

Lack of syphilis screening and delays in turnaround time of syphilis test results and treatment may lead to adverse pregnancy outcomes in over 50% of untreated maternal syphilis cases [10]. These include stillbirth, early neonatal death, preterm or low birthweight infants and clinical manifestations of congenital syphilis [10]. In 2016, there were an estimated 661,000 cases of congenital syphilis and over 350,000 adverse pregnancy outcomes related to untreated maternal syphilis worldwide [11]. Congenital syphilis is a preventable disease, as a single dose of benzathine penicillin G (BPG) administered at least four weeks prior to delivery is adequate to treat vertically transmitted infection. The World Health Organization (WHO) has stated that elimination of mother-to-child transmission (EMTCT) of syphilis is one of the most cost-effective public health interventions [12], and proceeded to define country-level processes and impact indicators for EMTCT of HIV and syphilis [13]. These include antenatal screening and treatment coverage of *≥* 95% and annual congenital cases rates of *≤* 50 per 100,000 livebirths for both HIV and syphilis [13]. An accurate, cost-effective dual HIV/syphilis rapid point-of- care test (POCT) would facilitate on-site screening for both infections in women who self-report an HIV-negative or unknown status at the time of presentation to antenatal care [7, 14]. In 2019, WHO issued a recommendation that rapid dual HIV/syphilis POCTs could be used as the initial screening test in antenatal care [15]. Further evaluations are needed to understand how best to support the introduction and scale-up of these assays at primary healthcare facilities across low- and middle-income countries (LMICs).

We sought to evaluate the clinical performance characteristics of two commercial HIV/syphilis POCTs compared to laboratory reference tests for the screening of maternal HIV and syphilis at primary healthcare centres (PHCs) in South Africa and Zambia. These POCTs are qualitative immunochromatographic lateral flow tests that simultaneously detect HIV and specific treponemal antibodies in human serum, plasma and whole venous or capillary fingerprick blood [16]. The purpose of a clinic-based evaluation is to determine how well POCTs perform as active case detection tools in real life settings. Although WHO has pre-qualified several dual HIV syphilis POCTs using performance thresholds of 98% sensitivity and 99% specificity for HIV and 85% sensitivity and 95% specificity for syphilis (treponemal) against reference laboratory standards in a laboratory-based evaluation using serum or plasma samples and performed by trained technicians, a decrease in performance is expected for field evaluations where whole blood from finger-pricked samples were used and the tests were performed by healthcare workers with limited training outside of controlled laboratory settings [17]. Secondary objectives were to determine feasibility and acceptability for patients, and operational characteristics of both assays including ease of implementation and acceptability to end-users (healthcare workers).

## Methods

### Study design and settings

Observational cross-sectional clinic-based evaluation of HIV/syphilis POCTs using a standardised study protocol developed by WHO, as previously described [16, 18]. This constitutes a pooled analysis using the individual participant data meta-analysis (IPD-MA) framework across five sites in two African countries implementing the WHO antenatal HIV and syphilis POCT evaluation. In South Africa, study sites included antenatal clinics at two PHCs in two different provinces, i.e. East Boom Community Health Centre, Pietermaritzburg, Kwa-Zulu Natal province (urban facility) and Kabokweni Clinic, White River, Mpumalanga province (rural facility). In Zambia, the study was conducted at three antenatal clinics in urban PHCs (Kamwala, Chipata and Chawama) in Lusaka. Individual participant data from Zambia on participant demographics and performance characteristics of the two POCTs were contributed to the ProSPeRo study [18].

### Study participants and procedures

Inclusion criteria included women aged 18 years and older at any stage of pregnancy whose HIV status was self-reported as being negative or unknown, provided they were able and willing to provide informed consent to participate in the study. Exclusion criteria included women younger than 18 years of age, those with a known HIV positive status, and those who were unwilling to consent to participate in the study. Eligible participants were enrolled consecutively following informed consent. Each participant was assigned a unique study identification number, which was delinked from all personal identifiers. A standardised paper-based questionnaire was administered by a study nurse to capture information on demographic characteristics (age, gestational age), clinical characteristics (past medical history i.e. recent antibiotic use in past 3 weeks and history of HIV/syphilis testing and treatment, genital ulcer disease at presentation), and feasibility and acceptability for study participants (proximity to clinic, preferences for stand-alone or dual HIV/syphilis testing, and willingness to wait for results).

A separate provider questionnaire was completed at the end of the study period by clinic nurses who performed the two POCTs. These collected information on ease of POCT use and result interpretation, time to result, as well as training time and number of tests required for competence/proficiency.

### Specimen collection and point-of-care testing

Prior to the commencement of clinic-based evaluations in South Africa, formal training was conducted by a team from the WHO and the South African National Institute for Communicable Diseases (NICD) to ensure that clinic nurses were proficient in use of POCTs and result interpretation.

In South Africa, the evaluations were conducted between 3 July 2018 and 31 December 2019. Two POCTs, i.e. SD Bioline HIV/Syphilis Duo (Abbott Diagnostics, IL, USA) and DPP® HIV-Syphilis (Chembio Diagnostic Systems Inc, NY, USA) were performed according to manufacturers’ instructions using capillary fingerprick whole blood. For each participant one clinic nurse performed both POCTs in tandem and recorded syphilis/HIV results (Reader 1). Subsequently, a second clinic nurse (blinded to result interpretation by first staff member) read and recorded results of both POCTs (Reader 2). Additionally, the second reader also recorded the results of the Chembio Micro Reader. All POCT results were read within the window of time recommended by manufacturers. In Zambia, the field evaluation was conducted from 1 September 2014 through 30 June 2015, as previously described by Kasaro MP et al. [18], using SD Bioline HIV/Syphilis Duo (Standard Diagnostics, Korea) and the DPP® HIV-Syphilis assay (Chembio Diagnostic Systems Inc, NY, USA).

### Reference testing

Study nurses collected approximately 10 ml venous blood from each participant in a serum-separator tube for reference laboratory testing. These specimens were kept refrigerated on site, and transported on ice to a central reference laboratory within 24 h of collection (i.e. the Sexually Transmitted Infections (STI) Reference Laboratory at the Centre for HIV & STI, NICD in Johannesburg, South Africa, and the Centre for Infectious Disease Research Laboratory in Lusaka, Zambia). Prior to testing at both reference laboratories, serum separation was done by centrifugation at 1,400 relative centrifugal force (RCF) for 10 min. The syphilis reference standard used in both countries was a specific treponemal assay, i.e. the *Treponema pallidum* particle agglutination (TPPA) test [Serodia-TPPA, Fujirebio Inc, Tokyo, Japan], and all TPPA-reactive specimens from South Africa were subsequently titrated. Additionally, a qualitative RPR assay [Immutrep® RPR assay, Omega Diagnostics Ltd, Alva, UK] was performed on all serum specimens (regardless of TPPA result), and reflexed to quantitative if reactive. In Zambia, initial syphilis screening was done using TPPA (without subsequent titration), and only TPPA-reactive specimens underwent further qualitative +/- quantitative RPR-testing.

In South Africa, two HIV 4th and 3rd generation qualitative enzyme immunoassays (Architect HIV Ag/Ab Combo, Abbott laboratories, Wiesbaden, Germany, and Genscreen HIV1/2 version 2, Bio-Rad laboratories, California, USA, respectively) were performed in parallel, with Western blot (Bio-Rad Laboratories, California, USA) used as the tie-breaker for any discordant antibody results between the two assays. In Zambia, two HIV POCTs were used as the reference standards, i.e. *Determine*™ *HIV*-1/2 (Abbott Diagnostics, Japan) and UniGold (Trinity Biotech, USA) with Western blot (Bio-Rad Laboratories, California, USA) used for resolution of discordant results. Clinical information and POCT results were not available to laboratory staff performing reference testing.

At each antenatal facility, participants received standard-of-care screening and treatment for both infections, based on national PMTCT guidelines.

### Data analysis

Sample size calculation was based on estimated performance of POCTs and expected antenatal seroprevalence of HIV and syphilis in women presenting to sentinel PHCs (obtained from the most recent national & provincial serosurveillance data available at the time) [19]. The formula used is contained in the 2006 WHO/TDR expert panel document on the evaluation of new diagnostic methods and techniques [20]. Estimated sensitivity of POCTs was as follows: HIV: 92%, syphilis (treponemal): 85%. For a confidence interval of +/- 5% around the point estimates of sensitivity and specificity, the target indicative recruitment for all sites was 1960 subjects. For syphilis, assuming 10% treponemal antibody prevalence and 85% POCT sensitivity, it was estimated that at least 195 positive cases would be needed. An HIV prevalence of 44% and a treponemal antibody prevalence of 10% was assumed in the study population at the Kwa-Zulu Natal site [19]. An HIV prevalence of 35% and a treponemal antibody prevalence of 10% was assumed in the study population in Mpumalanga [19]. As the recruitment of syphilis (treponemal positive) cases in South Africa was lower than expected, data from South Africa and Zambia were pooled within the IPD-MA framework to estimate POCT performance characteristics with greater precision. POCT results documented by Reader 1 were used in all analyses, as this would more closely approximate a real-world setting. Concordance between readers was measured by percentage of agreement and Cohen’s kappa coefficient. Sensitivity and specificity estimations were based on meta-analysis of pooled data from both countries using a bivariate model with random effects to account for within and between sites heterogeneity. In this model, sensitivity and specificity were jointly modelled to account for their intrinsic associations, as well as positive and negative likelihood ratios (LR + and LR-). Heterogeneity analysis was performed from the random effects model output by examining the random effects and testing significance of between-site variability. In addition, estimates for fixed effects models are provided. Various prevalence scenarios were proposed due to the known meta-analytic approach limitation on evaluation of pooled estimations for prevalence-dependent quantities. Positive and negative predictive values for POCTs were then calculated for each prevalence scenario for both HIV and syphilis, based on minimum and maximum prevalence of these infections by reference testing at study sites. Additionally, POCT sensitivity and specificity was ascertained for cases of probable active syphilis (using RPR *≥* 8 as a proxy) and for specimens with TPPA titres *≥* 2560 (median TPPA titre, South African data only). South African data were analysed in greater detail for outliers, i.e. POCT-negative specimens that showed high reactivity by RPR (*≥* 8). For each analysis, the denominator is representative of available data.

### Ethical considerations

The study protocol was independently peer-reviewed and approved by the Research Project Review Panel (RP2) of the WHO Department of Sexual and Reproductive Health and Research and the WHO Ethics Review Committee. In South Africa, ethical approval was obtained from the national and provincial departments of health, as well as the University of KwaZulu Natal Biomedical Research Ethics Committee (Ref: BE656/17) and the University of the Witwatersrand Human Research Ethics Committee (Ref: M171058). In Zambia ethical oversight was by the University of Zambia Biomedical Research Ethics Committee (Ref: REC 005-02-14), and the University of North Carolina at Chapel Hill Institutional Review Board (Ref: IRB 14–0528).

## Results

### Demographic and clinical characteristics

Data from a total of 3,412 participants enrolled in both countries were analysed. Table 1 lists the demographic characteristics of participants. The median age of women was similar in South Africa and Zambia, and most participants were in their second trimester of pregnancy at the time of study enrolment. Clinical characteristics are presented in Table 2 for South African participants only. More than 50% of these women said they had not previously been tested for syphilis, and 15% were unaware whether syphilis testing had been performed. The majority (> 90%) disclosed that they had received an HIV test; of these, 70% reported testing within the preceding 12-month period. Only 1 participant (who should not have been enrolled) self-reported a positive HIV infection status. A small minority (2%) had symptoms of genital ulcer disease, and less than 10% gave a history of recent antibiotic use. Hence, the majority of women who were both RPR- and TPPA-positive were asymptomatic and would therefore be diagnosed as having latent syphilis; approximately 40% of these were considered to have probable active syphilis (RPR *≥* 8).

**Table 1:** Demographic characteristics of participants, South Africa and Zambia

Characteristic*	South Africa (*n* = 1268)	Zambia (*n* = 2144)
Age [n; median (IQR)]	1268; 26 (22-30)	2123; 25 (21–30)
Age category [n (%)]		
• 15–19	104 (8.2)	238 (11.2)
• 20–24	413 (32.6)	744 (35.0)
• 25–34	631 (49.8)	927 (43.7)
• >/=35	120 (9.5)	214 (10.1)
Pregnancy Trimester [n (%)]		
• First	555 (43.8)	173 (8.1)
• Second	607 (48.0)	1705 (80.2)
• Third	104 (8.2)	248 (11.7)
• Missing	2	18

*denominators do not include missing data

**Table 2:** Clinical Characteristics of participants (South Africa only, *N* = 1,268)

Characteristics	*n* (%)
Previously tested for syphilis	
• Yes	334 (26.3)
• No	647 (51.0)
• Not aware	287 (22.6)
Previously diagnosed with syphilis (*N* = 334)	
• Yes	2 (0.6)
• No	322 (96.4)
• Not aware	10 (3.0)
Previous treatment with antibiotics in the past 3 weeks	
• Yes	108 (8.5)
• No	1,148 (90.5)
• Don’t know	12 (0.9)
Previously tested for HIV (*N* = 1,267)	
• Yes	1,191 (94.0)
• Last test < 1 year ago	829 (69.6)
• Last test >/= 1 year ago	362 (30.4)
Result of last HIV test (*N* = 1190)	
• Negative	1189 (99.9)
• Positive	1 (0.1)
Genital Sore/Ulcer (*N* = 1,266)	
• Yes	31 (2.4)
• No	1,235 (97.6)

### Performance characteristics

Table 3 depicts the pooled performance characteristics of the two POCTs for HIV and syphilis compared to the laboratory reference assays. In total, valid results were available for 3,361 syphilis and 3,353 HIV reference tests. The prevalence of TPPA-positivity and HIV infection at the study sites ranged from 3.7 to 9.9% (*n* = 253) and 17.8–21.3% (*n* = 643), respectively. The median RPR titre in TPPA-positive participants was 8 (interquartile range (IQR): 4–16; range: 1–128). In total 55 participants were syphilis and HIV co-infected (i.e. both RPR and HIV positive). The median TPPA titre in syphilis-exposed participants (South African data) was 2560 (IQR: 1280–10,240; range: 320–20,480). The agreement of testing results as read by two independent readers was high for both Bioline and Chembio with kappa statistics of 0.93 (95% CI = 0.91–0.94) and 0.91 (95% CI = 0.89–0.93) respectively for HIV and of 0.88 (95% CI = 0.85–0.92) and 0.87 (95% CI = 0.84–0.91) for syphilis.

**Table 3:** Performance characteristics of POCTs for syphilis and HIV compared to reference assays (random effects meta-analysis model using pooled data from South Africa and Zambia)

**BIOLINE**	**HIV**							**syphilis**							
Sites	I	P	N	FP	FN	Sensitivity (%)	Specificity (%)	I	P	N	FP	FN		Sensitivity (%)	Specificity (%)
South Africa 1	0	103	488	5	11	89.91 (82.66-94.85)	98.96 (97.60-99.66)	0	19	572	6	9		59.09 (36.35-79.29)	98.95 (97.72-99.61)
South Africa 2	2	130	492	15	4	96.64 (91.62-99.08)	97.02 (95.13-98.32)	2	21	604	2	8		70.37 (49.82-86.25)	99.67 (98.80-99.96)
Zambia 1	0	143	597	12	9	93.57 (88.15-97.02)	98.00 (96.53-98.96)	0	62	683	10	17		75.36 (63.51-84.95)	98.52 (97.30-99.29)
Zambia 2	0	123	550	16	13	89.17 (82.19-94.10)	97.11 (95.34-98.34)	0	59	613	13	21		68.66 (56.16-79.44)	97.85 (96.35-98.85)
Zambia 3	0	181	519	46	14	90.60 (84.74-94.77)	91.65 (89.02-93.82)	0	67	634	31	31		53.73 (41.12-66.00)	95.11 (93.13-96.65)
Fixed Effects Model	2	680	2646	94	51	91.99 (89.25-94.09)	96.50 (95.62-97.21)	2	228	3106	62	86		65.87 (58.94-72.19)	97.99 (97.34-98.48)
**Meta-analysis - Random Effects Model (Site is random)**						**92.08 (89.36-94.16)**	**97.17 (94.83-98.47)**							**65.99 (57.71-73.39)**	**98.48 (96.82-99.28)**
Between sites variability - SD (*p*-value)						7.11 (0.7936)	7.78 (0.1774)							7.18 (0.4760)	8.02 (0.2133)
DOR						399.31 (192.37-828.85)								125.65 (54.63-289.02)	
LR+						32.54 (17.64-60.03)								43.40 (20.44-92.16)	
LR-						0.08 (0.06-0.11)								0.3454 (0.2737-0.4358)	
**CHEMBIO**	**HIV**							**syphilis**							
Sites	I	P	N	FP	FN	Sensitivity (%)	Specificity (%)	I	P	N	FP	FN	Sensitivity (%)		Specificity (%)
South Africa 1	0	101	490	7	15	86.24 (78.32-92.09)	98.55 (97.03-99.41)	0	19	572	6	9	59.09 (36.35-79.29)		98.95 (97.72-99.61)
South Africa 2	1	138	485	27	8	93.28 (87.18-97.05)	94.64 (92.30-96.44)	1	22	604	3	8	70.37 (49.82-86.25)		99.50 (98.54-99.90)
Zambia 1	0	149	591	15	6	95.71 (90.91-98.41)	97.50 (95.91-98.59)	0	62	684	6	13	81.16 (69.94-89.57)		99.11 (98.08-99.67)
Zambia 2	0	125	549	18	13	89.17 (82.19-94.10)	96.75 (94.91-98.06)	0	58	614	12	21	68.66 (56.16-79.44)		98.02 (96.56-98.97)
Zambia 3	0	164	536	28	13	91.28 (85.54-95.27)	94.92 (92.74-96.60)	0	49	652	12	30	55.22 (42.58-67.40)		98.11 (96.72-99.02)
Fixed Effects Model	1	677	2651	95	55	91.37 (88.54-93.54)	96.47 (95.59-97.18)	1	210	3126	39	81	67.86 (60.98-74.03)		98.74 (98.20-99.11)
**Meta-analysis - Random Effects Model (Site is random)**						**91.46% (88.19-93.89)**	**96.71% (95.08-97.82)**						**67.93 (58.21-76.31)**		**98.79 (98.15-99.21)**
Between sites variability - SD (*p*-value)						7.18 (0.6223)	7.35 (0.2836)						7.29 (0.3509)		7.23 (0.5365)
DOR						315.11 (18.72-41.35)							172.77 (94.71-315.18)		
LR+						27.82 (18.72-41.35)							56.09 (35.89-87.66)		
LR-						0.09 (0.06-0.12)							0.33 (0.24-0.43)		
**CHEMBIO Microreader**	**HIV**							**syphilis**							
Sites	I	P	N	FP	FN	Sensitivity (%)	Specificity (%)	I	P	N	FP	FN		Sensitivity (%)	Specificity (%)
South Africa 1	0	113	478	9	5	95.41 (89.62-98.49)	98.13 (96.49-99.14)	0	23	568	8	7		68.18 (45.13-86.14)	98.59 (97.25-99.39)
South Africa 2	0	142	475	27	4	96.64 (91.62-99.08)	94.58 (92.21-96.40)	0	28	592	9	8		70.37 (49.82-86.25)	98.48 (97.14-99.30)
Fixed Effects Model	0	255	953	36	9	96.05 (90.44-98.43)	96.33 (94.24-97.68)	0	51	1160	17	15		69.39 (48.94-84.28)	98.54 (97.16-99.25)
DOR						638.07 (222.07-1833.40)								152.67 (51.03-456.72)	
LR+						26.15 (16.58-41.24)								47.43 (23.12-97.29)	
LR-						0.04 (0.02-0.10)								0.31 (0.17-0.57)	

*I I*nconclusive, *P *True positive, *N *True negative, *FP *False positive, *FN *False negative, *DOR *Diagnostic odds ratio, *LR +/- *Positive/negative likelihood ratio

Pooled sensitivity for syphilis (treponemal) compared to the reference TPPA assay was 66.0% (95% CI 57.7–73.4) with SD Bioline and 67.9% (95%CI 58.2–76.3) with Chembio (Table 3). Pooled specificity for syphilis was above 98% for both POCTs. Site-specific sensitivity/specificity analyses are presented in Additional Files 1–2 for syphilis and HIV. The positive predictive values (PPVs) for syphilis using SD Bioline were 62.6% and 82.7% for syphilis prevalences of 3.7 and 9.9%, respectively, and the negative predictive values (NPVs) 98.7% and 96.3% **(**Table 4). For the same syphilis prevalence estimates, the PPVs with Chembio were 68.5% and 86.1%, and NPVs 98.8% and 96.5%, respectively.

**Table 4:** Predictive values based on prevalence scenarios

	HIV					syphilis				
	Pooled Sensitivity	Pooled Specificity	Scenarios^a^	PPV	NPV	Pooled Sensitivity	Pooled Specificity	Scenarios^b^	PPV	NPV
BIOLINE	92.1%	97.2%	12.8%	82.7%	98.8%	66.0%	98.5%	1.7%	43.2%	99.4%
			17.8%	87.6%	98.3%			3.7%	62.7%	98.7%
			21.3%	89.8%	97.8%			9.9%	82.7%	96.3%
			26.3%	92.1%	97.2%			14.9%	88.4%	94.3%
CHEMBIO	91.5%	96.7%	12.8%	80.3%	98.7%	67.9%	98.8%	1.7%	49.6%	99.4%
			17.8%	85.8%	98.1%			3.7%	68.4%	98.8%
			21.3%	88.3%	97.7%			9.9%	86.1%	96.5%
			26.3%	90.8%	96.9%			14.9%	90.8%	94.6%
CHEMBIO Microreader (South Africa only)	96.1%	96.3%	12.8%	79.3%	99.4%	69.4%	98.5%	1.7%	45.4%	99.5%
			17.8%	85.0%	99.1%			3.7%	64.7%	98.8%
			21.3%	87.6%	98.9%			9.9%	84.0%	96.7%
			26.3%	90.3%	98.6%			14.9%	89.3%	94.8%

*PPV *Positive Predictive Value, *NPV *Negative Predictive Value based on random effects model

^a^Prevalence scenarios for HIV (min – max from study sites)

^b^Prevalence scenarios for syphilis (min – max from study sites)

When RPR *≥* 8 was used as the reference for probable active syphilis (101/253; 39.9%), SD Bioline sensitivity increased to 78.0% (95%CI 68.6–85.7). However, there was a slight corresponding reduction in specificity to 95.4% (95%CI 94.6–96.1). Similarly, Chembio sensitivity rose to 81.0% (95%CI 71.9–88.2), and specificity decreased to 96.0% (95%CI 95.3–96.7). The sensitivity of both POCTs for detecting specimens with TPPA titre *≥* 2560 i.e. the median TPPA titre (South African data) was 79.3% (95%CI 60.3–92.0), whereas the specificity was 55.0% (95%CI 31.5–76.9). Of 32 specimens for which both RPR and TPPA results were positive and titrated (South African data), there were 9 outliers (28.1%) with both POCTs. The TPPA titres for these specimens were as follows: 640 (*n* = 1), 1280 (*n* = 3), 5120 (*n* = 1), 10,240 (*n* = 1) and 20,480 (*n* = 3).

The pooled sensitivity of POCTs for HIV was as follows: for SD Bioline 92.1% (95%CI 89.4–94.2) and Chembio 91.5% (95%CI 88.2–93.9) (Table 3). Pooled specificity for HIV was 97.2% (95%CI 94.8–98.5) with SD Bioline and 96.7% (95%CI 95.1–97.8) with Chembio. At population-level HIV prevalences of 17.8% and 21.3%, the PPVs with SD Bioline were 87.6% and 89.8%, respectively (Table 4). PPVs with Chembio for the same HIV prevalences were 85.7% and 88.3%, respectively. The NPVs with SD Bioline were 98.3% and 97.8%, respectively. Similar NPVs were obtained with Chembio, i.e. 98.1% and 97.7%, respectively. Diagnostic sensitivity increased with the Chembio Micro Reader for both syphilis (69.4% ; 95% CI 56.5–82.3) and HIV (96.1%; 95% CI 93.5–98.6). The PPVs and NPVs which are based on various prevalence estimates were not significantly impacted.

### Feasibility and acceptability for patients

In South Africa, the median time spent in travelling to the clinic from the place of residence was 30 min (IQR: 15–30 min). The majority (98.8%; 1251/1266) stated that they would be willing to wait for results at the clinic; of those, 67.4% were willing to wait for up to 30 min or more for the results. The vast majority (91.4%; 1156/1264) preferred dual over single stand-alone HIV and syphilis POCTs. In Zambia, 99.7% of patients preferred the dual tests over single tests and 99.9% were willing to wait for the results; of those all reported willingness to wait up to 30 min or more for results.

### Operational characteristics

Clarity of kit instructions, ease-of-use, and ease-of-interpretation of results were reported to be ‘very clear’, ‘very easy’, ‘excellent’ or ‘unambiguous’ for over 75% of providers for SD Bioline, and were slightly lower (50%-60%) for Chembio. Most providers reported that test results were available in 30 minutes or less (93% and 90%), with hands on time of 5 minutes or less (100% and 70%) for SD Bioline and Chembio, respectively. For training time required, approximately one third (30%) of providers reported needing more than 1 hour for SD Bioline training, and two thirds (67%) for Chembio training (Additional file 3).

## Discussion

Data from South Africa and Zambia revealed a high prevalence of treponemal antibody positivity (> 5%) and HIV positivity (> 10%) in the study cohort. Most syphilis-positive women in our study were latently infected; and approximately 40% of these were considered to have probable active syphilis (RPR *≥* 8). While there is some overlap in the distribution of RPR titres across different stages of syphilis, the majority of persons having early latent syphilis are expected to have relatively high RPR titres [21, 22]. A quantitative maternal RPR titre *≥* 8 was therefore used as a marker of active infection in the mother with a higher risk of in-utero foetal infection and congenital syphilis [23, 24]. A proportion of HIV-infected women were co-infected with syphilis, and this would increase the risk of vertical HIV transmission [25] and adverse birth outcomes [26].

Our clinic-level data reveal that both POCTs have a relatively low sensitivity, but high specificity for syphilis. Sensitivity increased considerably to almost 80% when a proxy of RPR *≥* 8 was used to define “active syphilis” and an increased risk of vertical transmission. Both POCTs showed moderate sensitivity for HIV and high specificity. A systematic review and meta-analysis of 18 studies evaluating three dual HIV/syphilis POCTs, including the SD Bioline and Chembio assays, showed moderate-high sensitivity (93.8–100%) and specificity (94.2–100%) for HIV, and lower but largely acceptable performance characteristics for syphilis (sensitivity: 47.4–100%; specificity: 90.8–100%) [27]. These studies were conducted using whole blood samples from a variety of at-risk populations as well as archived serum specimens, and included both field and laboratory evaluations. A laboratory evaluation of specimens from Baltimore STI clinic attendees using SD Bioline HIV/Syphilis showed approximately 92% sensitivity and 99.5% specificity for HIV, when compared to an imperfect reference standard (either oral fluid HIV antibody testing performed by clinic personnel or information obtained from chart review); and a lower sensitivity for TPPA-positive syphilis (69.7%), which increased to 85.7% when comparing RPR positive specimens [28]. A study of over 4,500 women with an overall HIV prevalence of 3% at 12 ante-natal clinics in Nigeria showed good positive and negative agreements for HIV on SD Bioline HIV/syphilis compared to standard-of-care HIV POCTs [29]. Only four specimens were treponemal antibody and low-titre RPR positive, all four tested syphilis negative on the dual POCT; however the POCT specificity for syphilis was 99.9% with no false-positive results [29].

An essential component of the evaluation process is the estimation of positive and negative predictive values of POCTs, based on the prevalence of infection in target populations. Although the PPV for syphilis at our study sites was low-moderate with both POCTs, the NPV was relatively high. Similarly, although the PPV for HIV was below 90% for the range of HIV prevalences observed in the study populations, the NPV was approximately 98%. This suggests that these POCTs could be effectively incorporated into antenatal testing algorithms to significantly reduce peri-natal transmission of syphilis and HIV and prevent adverse pregnancy outcomes. The WHO recommends a 99% PPV for an HIV testing algorithm using a combination of tests with at least 99% sensitivity and 98% specificity [30]. The first test should have the highest sensitivity with subsequent tests having high specificity, and a three test algorithm used if HIV population prevalence is below 5% [30]. Our POCT results are similar to those observed in other field studies, which suggests that clinical performance characteristics in relatively resource-constrained real-world settings may differ from laboratory-based analytical performance characteristics, when assays are performed by busy healthcare workers using capillary whole blood [18, 29]. We observed a proportion of outliers who would be categorised as having active syphilis by reference testing, but had negative syphilis POCT results. A possible explanation, apart from user error, is the presence of excess serum antibody which would interfere with the binding of antibody to bound antigen on the POCT strip. This is described as a hook effect, and is similar to the prozone phenomenon observed with non-treponemal flocculation tests such as the RPR assay [31]. Healthcare workers need to be cognisant of the fact that even weakly-reactive (faint) test lines on these POCTs should be interpreted as positive results [32].

Prior to implementation and scale up of dual POCTs, it is important to consider the local context and conditions. Rapid tests are particularly useful at facilities that have high rates of loss to follow-up, late presentation to ante-natal facilities, and limited laboratory access, where a same-day actionable result would be preferred. Studies have shown that there is a trade-off between the accuracy and accessibility of diagnostic tests for syphilis, and a lower accuracy may be acceptable if it translates into a higher testing and treatment coverage required for EMTCT [33, 34]. In South Africa, there has been an increase in congenital syphilis clinical notifications reported via the NICD Notifiable Medical Conditions surveillance platform since July 2017 [35]. The most recent antenatal serosurvey conducted in 2019 revealed a syphilis screening coverage of 93% (when missing data were assumed to indicate a lack of screening), and an HIV screening coverage of 99.8% based on record review [3]. At the time of the survey, almost 19% of syphilis results were not on file. Approximately 96% of those with a documented HIV-positive status had been initiated on anti-retroviral therapy; however, treatment with at least one dose of BPG was recorded for only 85% of syphilis seropositive participants [3]. Testing only once in early pregnancy may miss at-risk women who are infected from untreated partners in late pregnancy [36]. There are anecdotal and published case reports of congenital syphilis arising from missed opportunities for re-screening at-risk women in the third trimester of pregnancy [37]. This would include a proportion of women presenting with early primary syphilis at the first antenatal visit, who have RPR seronegative or low-titre specimens that may test negative with the initial treponemal POCT.

Large-scale implementation should involve the systematic integration of POCTs into existing maternal and child health programmes, and incorporation into existing national screening algorithms [15]. It is important to ascertain that POCTs have been assessed by internationally-recognised regulatory authorities, and that internal and external quality assurance procedures are adhered to. SD Bioline HIV/Syphilis is on the WHO list of pre-qualified in-vitro diagnostic products [38]. Although not pre-qualified by WHO, Chembio DPP® HIV-Syphilis has been approved by the United States Food and Drug Administration [39]. Numerous countries including those in this study are staging for implementation of these multiplex tests [40]. A dual POCT may be used to exclude HIV and syphilis at the first antenatal visit; as well as repeated at 32–34 weeks if the first antenatal test is negative for both infections [15]. A stand-alone HIV POCT may be used in HIV screening at each of the other routine basic antenatal care visits, as per South African prevention of mother-to-child transmission PMTCT guidelines [9]. In keeping with our findings, and the existing standard-of-care, HIV positivity on a dual HIV/syphilis POCT should be confirmed by a different single HIV POCT [6, 30]. A syphilis positive result on dual POCT test should prompt treatment initiation with a single dose of BPG, and reflex confirmatory testing with laboratory-based treponemal and non-treponemal assays, if available [41]. The completion of the three-dose penicillin regimen for latent syphilis should ideally be based on a confirmatory RPR result, better indicating the presence of active syphilis. A systematic review on the impact of syphilis rapid diagnostic testing at antenatal sites already conducting HIV POCTs has shown increases in both syphilis and HIV screening coverage in LMIC settings [42]. A feasibility study conducted in Zambia and Uganda demonstrated that introduction of antenatal syphilis POCT significantly improved syphilis screening and treatment coverage in HIV-infected pregnant women, without compromising HIV care [43]. We do not have sufficient data to comment on the use of dual HIV/syphilis POCTs for syphilis screening in pregnant women with a known HIV-positive status, as this was not evaluated in our study.

Policy-makers in resource-constrained settings require evidence of the economic impact of any new intervention. A modelling of the cost-effectiveness of dual HIV/syphilis POCTs in four countries with varying prevalence of infection, including two in sub-Saharan Africa (Kenya and South Africa), showed a cost benefit for all countries when they were routinely used for screening at the first antenatal visit, as well as for retesting in the late antenatal period [14]. Simulated modelling of the cost-effectiveness of dual HIV/syphilis POCT use among 100,000 pregnant women in Malawi showed that this screening strategy was the least costly, and resulted in the fewest adverse pregnancy outcomes, compared to single on-site or laboratory screening tests for both, or rapid screening for HIV only [44].

We found that dual POCTs were largely preferred by pregnant women attending PHCs, and that women would be willing to wait for results based on their turnaround time. Other field studies have also reported good uptake of dual HIV/syphilis POCTs among pregnant women attending both urban and rural antenatal care facilities [29, 45]. Clinic nurses who performed and evaluated POCTs gave mostly favourable feedback with respect to their utility at point-of-care. A relatively longer period of training and hands-on-time for testing was needed for the Chembio assay, possibly due to the additional buffer and steps required for this POCT. It would therefore be feasible to introduce these assays into testing algorithms; however, there are several operational requirements. These include a programme for continuous training at PHCs with high staff turnovers, to address and monitor user-related issues such as the addition of correct reagents, time to reading of results and correct interpretation of test lines. In Ghana, various operational challenges for syphilis POCT implementation in antenatal care were identified, such as a lack of sustained healthcare worker training, testing protocols, programme supervision and adequate record keeping [46]. Participation in an external quality assurance programme should be prioritised; this can be administered by a national reference laboratory, using dried tube test panels with regular feedback and institution of corrective action if necessary [47, 48]. The establishment of a reliable procurement system and supply chain with stock control will ensure that screening coverage is not compromised by stock-outs of test kits [49]. Additionally, clear communication on syphilis testing and immediate treatment must be provided by healthcare providers to pregnant women in order to facilitate effective utilisation of dual POCTs. It is notable that the majority of women in our study at South African sites confirmed that they had been tested for HIV; however, more than 75% reported that they had either not been screened for syphilis or were unaware of their screening status, most likely due to lack of information. Ultimately, the success of the programme will be contingent on impact indicators such as the treatment coverage of syphilis and HIV seropositive women, and the ready availability and accessibility of drugs such as BPG. A recent global shortage of BPG, which affected supply in both South Africa and Zambia, was attributed to a shortage of the active pharmaceutical ingredient, as well as to quality assurance issues related to manufacture [50]. Forecasting of BPG volumes needed to scale-up antenatal implementation of dual HIV/syphilis POCTs, and meet an EMTCT target of 95% in 11 focus countries, showed an increased demand of 160% by 2030 [51]. This points to a need for programmes to plan for BPG scale-up alongside implementation of dual HIV/syphilis POCTs [52, 53]. Finally, a method of integrating data from antenatal care facilities into existing information systems will be required for evaluating national progress towards EMTCT, and for reporting process and impact indicators to WHO.

Strengths of our study include the large sample size of participants and POCT evaluation data from multiple centres and settings using standardised WHO core protocols. Limitations include the fact that POCT and/or reference test results were unavailable for approximately 2.5% participants. Additionally, the relatively small sample size of maternal syphilis with lower-than-expected treponemal antibody prevalence in the sample led to imprecise POCT syphilis sensitivity/specificity estimates with wide confidence intervals.

## Conclusion

Our findings on the performance and operational characteristics of dual HIV/syphilis POCTs show that they may be effectively incorporated into existing antenatal screening algorithms within the local context, in order to improve syphilis screening coverage in pregnant women. We also highlight the importance of adequate training and supervision of field workers performing POCTs, and the need for a quality management system to monitor performance indicators. These data will be useful to policy-makers and programme managers as they strive towards achieving national EMTCT targets for HIV and syphilis.

### Supplementary Information

Supplementary Material 1:  Fig. 1. Performance Characteristics of dual HIV/syphilis POCTs for syphilis compared to reference TPPA assay (site-specific data).

Supplementary Material 2:  Fig. 2. Performance Characteristics of dual HIV/syphilis POCTs for HIV compared to reference assays (site-specific data).

Supplementary Material 3:  Fig. 3. Operational Characteristics of dual HIV/syphilis POCTs.

## Data Availability

We will be following the WHO research data sharing policy (link: *Sharing and reuse of health-related* data *for research purposes: WHO policy and implementation guidance*), with the POCT Editorial Board providing governance for review of any data requests. As per the WHO guidelines, we will be able to share on the Zenodo research repository platform the process to request an anonymised dataset, and metadata for this study on request.

## Abbreviations

BPG: Benzathine penicillin G
DOR: Diagnostic Odds Ratio
DPP: Dual Path Platform
DTS: Dried Tube Specimens
EMTCT: Elimination of mother-to-child transmission
HIV: Human Immunodeficiency Virus
LMICs: Low- and middle- income countries
LR: Likelihood ratio
MR: Micro Reader
NICD: National Institute for Communicable Diseases
NPV: Negative Predictive Values
PHC: Primary healthcare centre
POCT: Point-of-Care Test
PPV: Positive Predictive Values
PMTCT: Prevention of mother-to-child transmission
ProSPeRo: Project on Sexually Transmitted Infection Point-of-Care Testing
RCF: Relative centrifugal force
RP2: Research Project Review Panel
RPR: Rapid Plasma Reagin
TDR: Special Programme for Research and Training in Tropical Diseases
STIs: Sexually Transmitted Infections
TPPA: Treponema pallidum Passive Particle Agglutination
WHO: World Health Organization
