# Supplementary material for: Clinic-based evaluation of point-of-care dual HIV/syphilis rapid diagnostic tests at primary healthcare antenatal facilities in South Africa and Zambia
Source: BMC Infect Dis. 2024 Jun 19;24(Suppl 1):600. doi: 10.1186/s12879-024-09463-1 (PMC11186134; doi:10.1186/s12879-024-09463-1)

**Additional file 1**

**Fig1: Performance Characteristics of dual HIV/ Syphilis POCTs for syphilis compared to reference TPPA assay (site-specific data)**


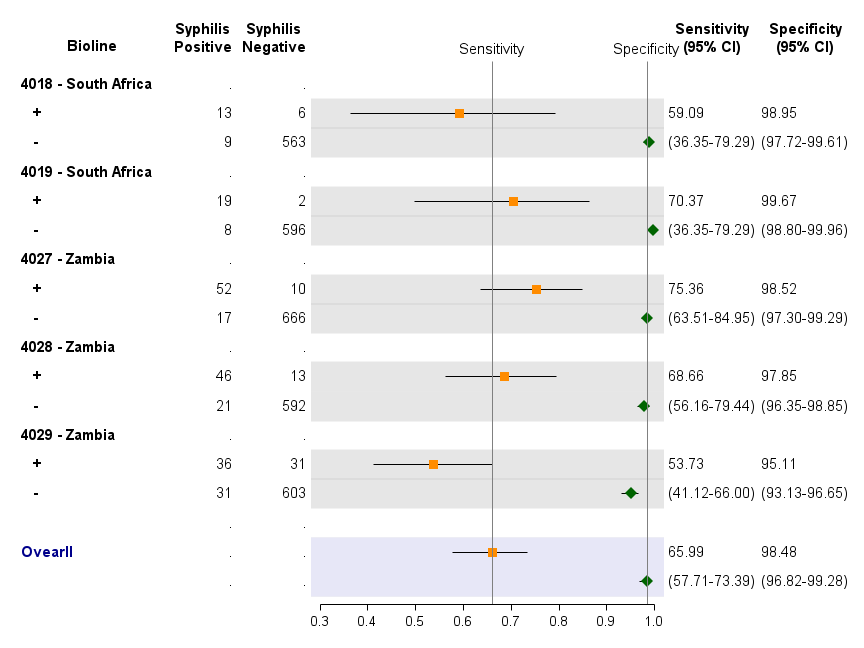


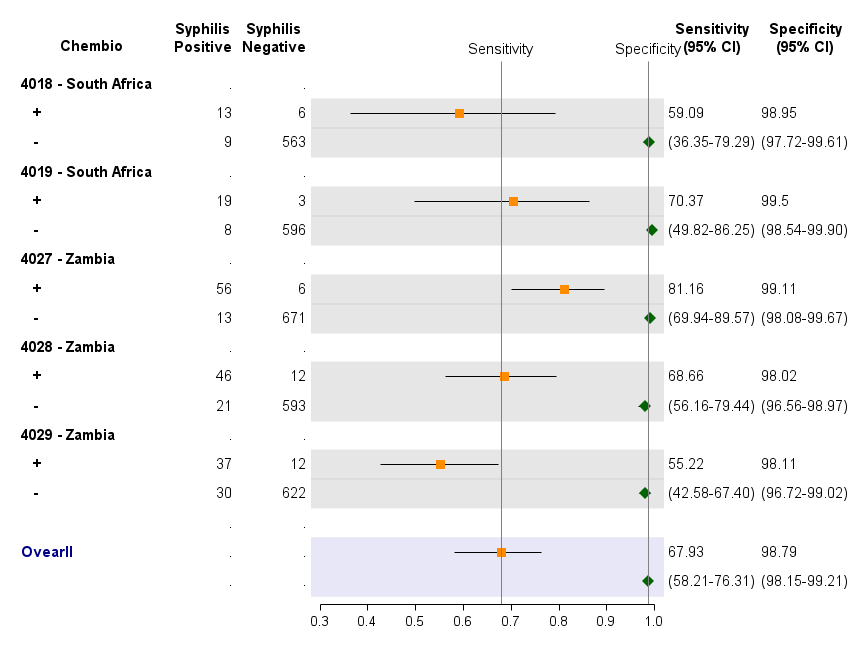

Supplement: Supplementary file 1 — Supplementary Material 1: Fig. 1. Performance Characteristics of dual HIV/syphilis POCTs for syphilis compared to reference TPPA assay (site-specific data). [file 12879_2024_9463_MOESM1_ESM.docx]
