# Supplementary material for: Clinic-based evaluation of point-of-care dual HIV/syphilis rapid diagnostic tests at primary healthcare antenatal facilities in South Africa and Zambia
Source: BMC Infect Dis. 2024 Jun 19;24(Suppl 1):600. doi: 10.1186/s12879-024-09463-1 (PMC11186134; doi:10.1186/s12879-024-09463-1)
Supplement: Supplementary file 2 — Supplementary Material 2: Fig. 2. Performance Characteristics of dual HIV/syphilis POCTs for HIV compared to reference assays (site-specific data). [file 12879_2024_9463_MOESM2_ESM.docx]

**Fig2: Performance Characteristics of dual HIV/ Syphilis POCTs for HIV compared to reference assays (site-specific data)**

**
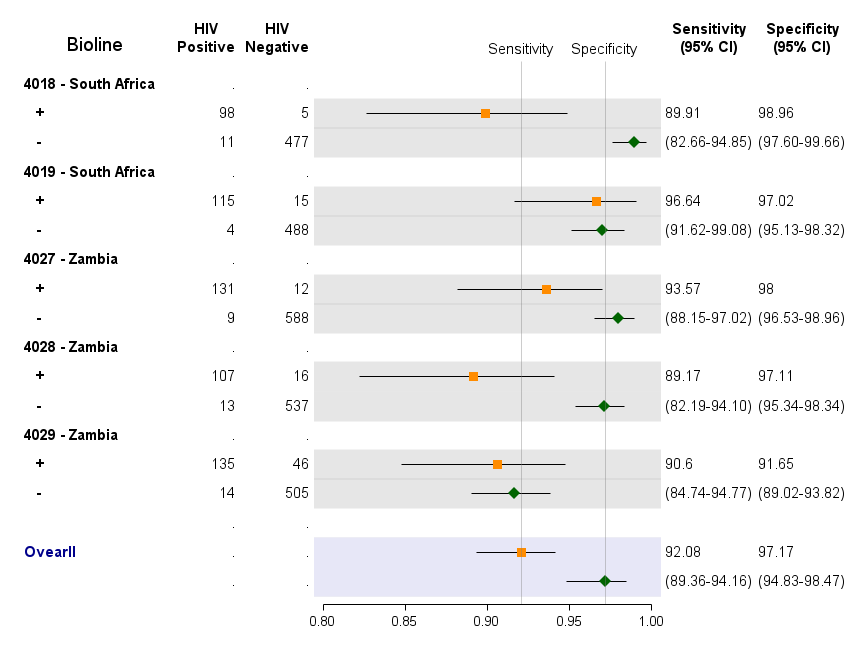
**

**
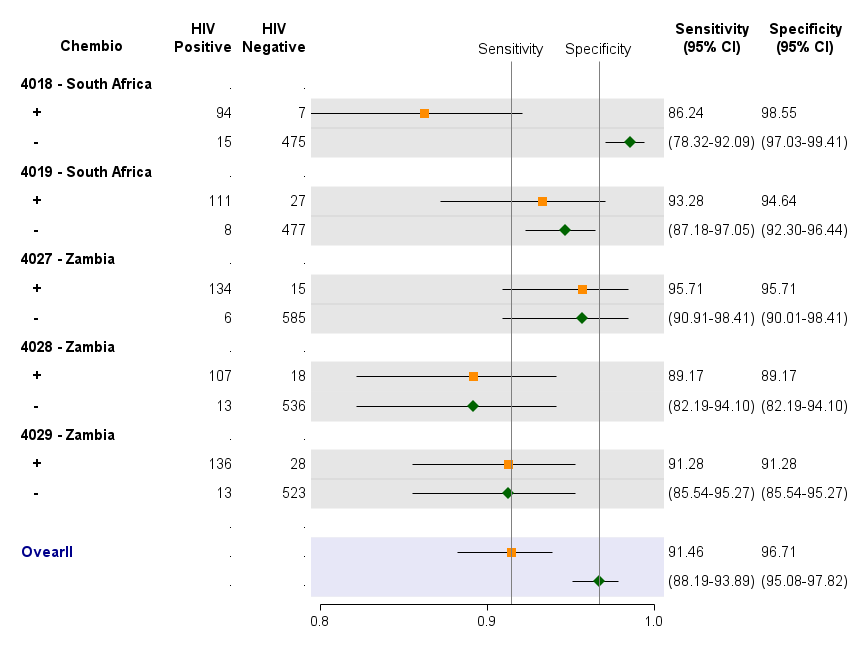
**
