# Supplementary figures and images for: Clinic-based evaluation of point-of-care dual HIV/syphilis rapid diagnostic tests at primary healthcare antenatal facilities in South Africa and Zambia
Source: BMC Infect Dis. 2024 Jun 19;24(Suppl 1):600. doi: 10.1186/s12879-024-09463-1 (PMC11186134; doi:10.1186/s12879-024-09463-1)

**Fig 3: Operational Characteristics of dual HIV/ Syphilis POCTs**


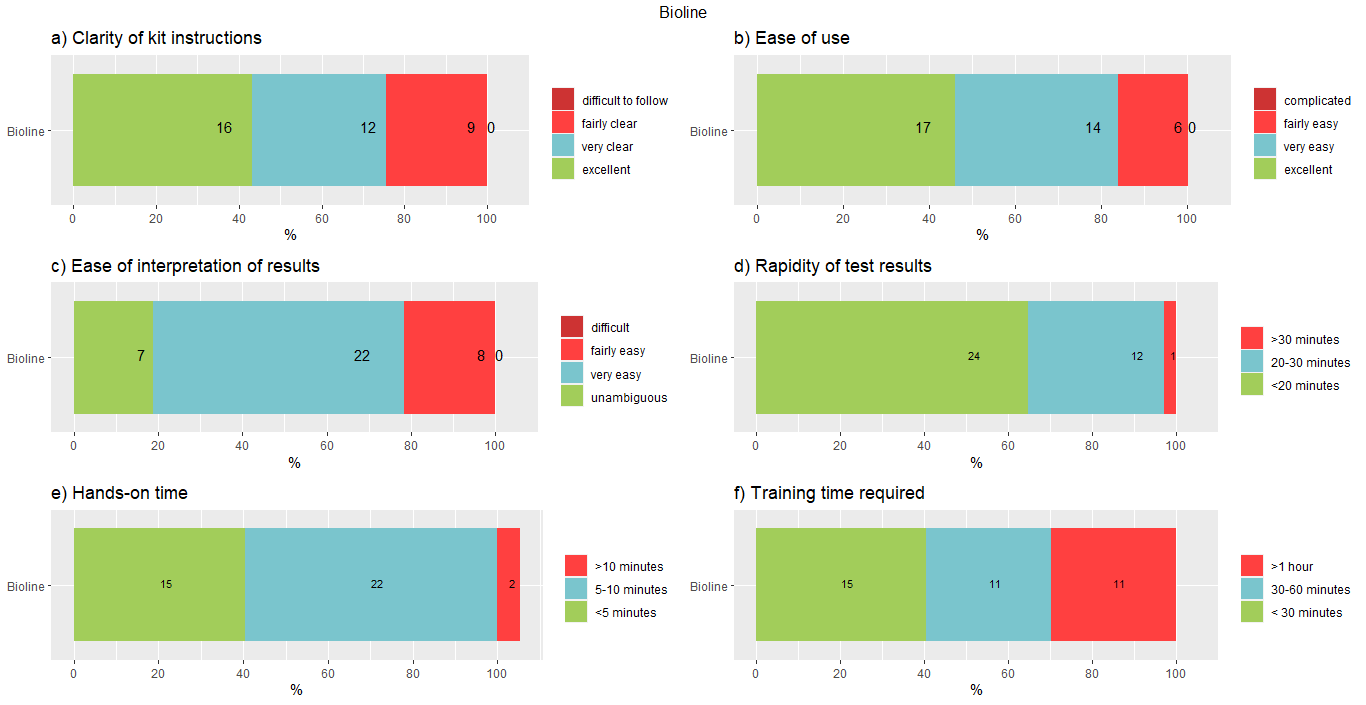


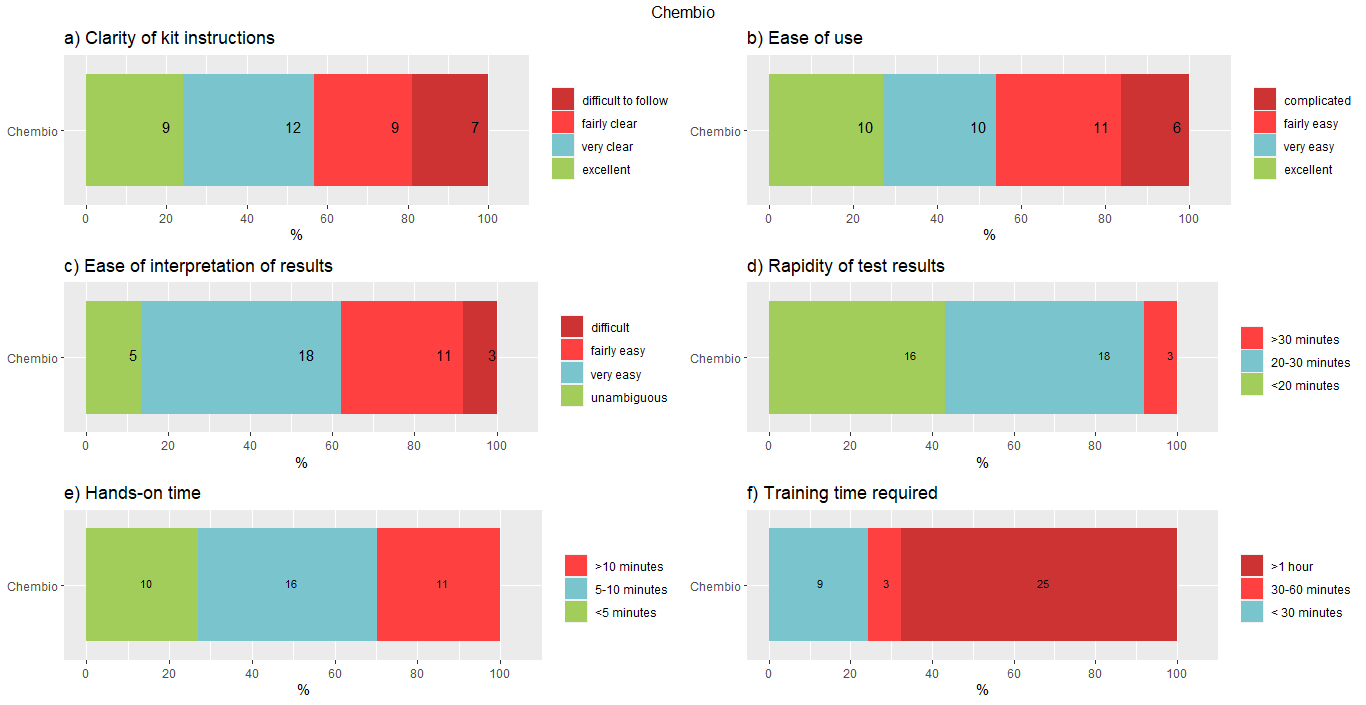

Supplement: Supplementary file 3 — Supplementary Material 3: Fig. 3. Operational Characteristics of dual HIV/syphilis POCTs. [file 12879_2024_9463_MOESM3_ESM.docx]
